# Phenomenology of psychiatric emergencies

**DOI:** 10.3389/fpsyg.2023.1212054

**Published:** 2023-09-12

**Authors:** Stefano Goretti, Cecilia Maria Esposito, Gilberto Di Petta

**Affiliations:** ^1^Department of Psychiatry, Torrecárdenas University Hospital, Almeria, Spain; ^2^Department of Brain and Behavioral Sciences, University of Pavia, Pavia, Italy; ^3^SPDC, Mental Health Department, Santa Maria delle Grazie Hospital, Naples, Italy

**Keywords:** psychiatric urgency, emergency room, phenomenology, psychosis, psychiatric urgencies

## Abstract

**Introduction:**

Psychiatric urgency is defined as a situation of serious mental suffering and behavioral alteration, which promptly requires adequate treatment; we talk about emergency when the condition can be life-threating. Even if until now neglected by phenomenological psychopathology, the emergency issue faces a clinical management challenge in which the phenomenological method becomes fundamental. The purpose of this manuscript is then to explore the phenomenological perspective of psychiatric emergencies. The manuscript is organized into four sections: the first deals with the encounter in clinical phenomenology, the second with the life-word of the crisis, the third with the atmosphere of emergency; finally, a final section on the importance of the phenomenological method for the clinician.

**The encounter in clinical phenomenology:**

The centrality of the encounter in clinical phenomenology cannot be stressed enough. It is not just the encounter between doctor and patient, but also and above all the encounter between two men, between two subjects. And it is in the affective space between them, in the intersubjectivity and intercorporeality of their encounter, that the transformative power of understanding emerges and reverberates from both sides. The approach to the other must be respectful, along the lines of the ethics of approximation, it must recognize the other as other and not overwrite it with one’s own prejudices. Otherwise, if clinicians are not sufficiently trained in the encounter, the risk is to get stuck in the anguish of the instant, to be absorbed by it, to become its tools. It is precisely the atmosphere of the emergency room that is full of expectations, haste, anxiety, which actually hinders the possibility of encountering. Instead, this possibility must be recovered, because the encounter is the founding aspect of every clinical interview, of every diagnostic suspicion, of every therapeutic resolution.

**The life-word of the crisis:**

Seizing the encounter in its immediacy and in its totality, through the atmosphere that characterizes it, means for the clinician to position himself not outside the crisis, in an observational position in front of the patient, but to position himself next to him, to immerse himself in his life-world. Only then will the explosiveness of his symptoms appear to us not only as a symptomatic cascade to be contained and extinguished, but as the expression of a life-world in crisis. To use Ey’s terminology, the madness of an instant must be placed within the madness of a lifetime. The patho-gnostic structures of the psychiatrist must tune into the structures of the life-world of the crisis, with the perspective of giving meaning, of helping the subject to re-inscribe the crisis within his history, and to overcome it.

**The atmosphere of emergency:**

The experience of emergency is in fact detached from daily life of our being-in-the-world, both from the clinician’s side and from that of the patient, who loses himself in this pathically charged and tense atmosphere and needs someone to walk alongside him to find the reins of his world. The context of the emergency room puts the clinician in the position of applying Strauss’s sympathetic perception of the world, made up of atmospheres, sensations, profiles, and not of eidetic knowledge. The concept of atmosphere, inaugurated by Tellenbach and taken up in recent years by several authors, appears fundamental in understanding the amalgam of emotional tension, haste and immersiveness that characterizes the emergency room environment. An atmosphere that can become oppressive, if not thematized, and that can lead the clinician to defend himself in the haste and superficiality of the intervention.

**The phenomenological method:**

Psychiatric crisis is always a situation in which we are thrown, perhaps to the highest degree, and the unfolding of references between the self and the world and between the self and the others becomes an essential skill. Even in the absence of an adequate setting, in the intersection between several pressures, the phenomenological method retains its panoramic gaze intact. We define it panoramic because it does not aim only at the observation and description of the present phenomena, which are generally characterized by violence, anguish, chaos. It is through the suspension of the *epochè* that the clinician can distance himself from the oppressive atmosphere of the crisis and grasp the coordinates of the patient’s life-world. Only with this attitude does an authentic encounter become possible even in the difficult situation of emergency, paving the way for the challenge of care.

## Introduction

Psychiatric urgencies are defined as situations of serious mental suffering and behavioral alteration, which promptly require adequate treatment; we talk about emergencies when the conditions can be life-threating. Their essential characteristics are therefore the severity, the need for intervention and his speed (Wheat et al., 2016). Faced with an urgency, it is first essential to exclude the organic causes that could have caused it, from hormonal and metabolic imbalances to acute neurological and inflammatory conditions (Schick et al., 2022). Subsequently, psychiatric disorders that can give rise to situations of urgency must be considered: psychomotor agitation, acute psychoses, manic episodes, suicidal intentionality and severe behavioral alterations (Salani et al., 2021).

Of all the situations that a psychiatrist has to manage in his clinical practice, that of the psychiatric emergency is certainly one of the most complex and controversial. On the one hand, because first aid tools are often not designed for the needs of the person suffering from mental disorders; on the other, because the pressure of urgency can invalidate the therapeutic relationship (Barra et al., 2007). Knowing psychopathology appears to be essential to discern between different clinical conditions which, in the explosiveness of urgency, may seem superimposable. In this sense, phenomenological psychopathology can be an accurate tool for understanding and identifying patients’ experiences (Stanghellini and Broome, 2014; Fuchs et al., 2019). Throughout its long history, it has allowed for accurate descriptions and the unfolding of the life-world of several psychopathological conditions (Stanghellini et al., 2018). It is therefore surprising how until now the situations of urgency has been neglected by phenomenological psychopathology. In fact, it is our opinion that precisely the condition of the emergency room, reducing the interaction to the I-you relationship and often emerging from a context that is not adequate to the indications of the external setting, makes the tools of the internal setting all the more essential (Cooper, 2019). The importance of a certain way of being with the patient is therefore underlined, regardless the context in which this occurs.

The purpose of this manuscript is then to explore the phenomenological perspective of psychiatric emergencies. The manuscript is organized into four sections: the first deals with the encounter in clinical phenomenology, the second with the life-word of the crisis, the third with the atmosphere of emergency; finally, a final section on the importance of the phenomenological method for the clinician.

### The encounter in clinical phenomenology

Clinical phenomenology has always paid particular attention to the theme of the encounter, understood as a nodal point of observation and understanding of psychopathology (Di Petta, 1996; Jaspers, 1997). The experience of the encounter is strongly transformative on both men who experience it, in the context of a relationship that would like to be symmetrical, not hierarchical (Di Petta, 1996). In fact, we develop our subjectivity starting from the encounter with otherness, which therefore has a founding power over ourselves (Kojève, 1969; Sartre, 2003). It is therefore not surprising that the importance of this transformative power has been underlined in psychopathology, in order not only to explain mental illnesses, but to understand the experience of the Other (Husserl, 1962). Every man brings his own wealth of experiences and otherness, which in the moment of confrontation with another human being is brought into play (Binswanger, 1957).

This dual dimension must never fail, not even when we realize that the man in front of us is suffering from a mental illness: recognizing the constellation of symptoms as a snapshot that allows us to make a diagnosis is an incontrovertible moment, but it does not exhaust the life experience of that person (Rossi Monti and D'Agostino, 2018). Clinical phenomenology has always been interested, rather than in finding frames, in understanding what it means for a given individual to have his own experience of the world and of the Self (Jaspers, 1997).

Understanding takes place within an I-you relationship, a relationship between men rather than between doctor and patient. It is not an intuitive, uncritical understanding, but a rigorous method that aims not to overwrite the experience of the Other with one’s own (Rashed, 2015). The *epoché*, or the suspension of judgment (and prejudice), becomes an essential tool for accessing the experiences of the Other (Binswanger, 1958; Husserl, 1962). We need to know how to eclipse our world, while remaining emotionally present, to welcome that of another into us, help him unfold it, experience it together. An essential element therefore becomes the *ethics of approximation*: respect for the difference of Other, in the awareness that we will never be able to exhaust his life-world experience (Stanghellini, 2016).

The richness of this perspective can be seen when the protections of the external setting are lacking and we are alone in front of our patient, as happens in the emergency room. Then, the method by which we approach the Other becomes the fundamental tool we have. The two most pregnant potentials of the encounter are represented by the unfolding of the patient’s life-world and by the recognition (Di Petta, 1996; Stanghellini, 2019). If unfolding allows us to place the patient’s experience in a grid of existential meaning, recognition conveys the acceptance of the Other’s subjectivity as radically other.

The event of the encounter takes place neither in my field nor in the field of the Other, but in an intermediate field between us, the field of intersubjectivity, of intercorporeity: a close distance, emotionally charged, from which new readings and new meanings for both parties emerge (Merleau-Ponty, 1962; Di Petta and Tittarelli, 2019). If we can speak of truth, this truth is placed between us, and thus all violence that reductionism imposes on our experience of the world is eliminated (Di Petta, 1996). As Binswanger would say, one heals from the *cancer* of the split between subject and object, which has affected both philosophy and psychiatry, towards the shared experience of being-in-the-world (Binswanger, 1947). The ethical value of this ontological setback, of this change of perspective, is evident: following Cargnello (1966), the difficulty in the psychiatrist’s work lies in the continuous tension between having-something-in-face and being-with-someone. The encounter represents the paradigmatic moment of this criticality, and the testing ground for each of us to learn to see the man beyond the sick.

The encounter training cannot be overlooked for a psychiatrist. Otherwise, the risk is to get stuck in the anguish of the instant, to be absorbed by it, to become its tools. It is precisely the atmosphere of the emergency room that is full of expectations, haste, anxiety, which hinders the possibility of encountering. Instead, this possibility must be recovered, because the encounter is the founding ground of every clinical interview, of every diagnostic suspicion, of every therapeutic resolution.

### The life-word of the crisis

The contribution of the phenomenological method, applied to the description of the fundamental structure of the acute psychiatric event, is crucial. It represents the rigorous attempt to grasp the crisis in its immediacy and in its totality, with a centered-person approach (Stanghellini and Aragona, 2016). The modal determinants of the crisis, i.e., how it arises, how it becomes worldly, and how it involves the clinician himself, are fundamental to grasp. They allow the clinician to move appropriately, to be able to operate on them to change them for a resolution.

Seizing the encounter in its immediacy and in its totality, through the atmosphere that characterizes it, means for the clinician to position himself not outside the crisis, in an observational position in front of the patient, but next to him, to immerse himself in his life-world (Cargnello, 1966). We should consider the event as a whole and favor the massive (sensory and “pathic”) involvement of the clinician in it, not as an extrinsic or incoming part, but as an active, acting and reacting part (embedded and embodied). This approach can favor the deployment of implicit devices of knowledge, which will enable the clinician and the patient to gain a way out of the crisis, through a special attunement and conferring meaning to it (Binswanger, 1947; Di Petta and Tittarelli, 2019). Only then will the explosiveness of his symptoms appear to us not only as a symptomatic cascade to be contained and extinguished, but as the expression of a life-world in crisis (Stanghellini and Rossi Monti, 2009). To use Ey’s terminology, the madness of an instant must be placed within the madness of a lifetime (Ey et al., 2010).

To achieve this goal, we need to understand the crisis within the *existentialia* that characterize it: how is the crisis expressed in the world (Van den Berg, 1955; Heidegger, 2008)? The patho-gnostic structures of the psychiatrist must tune into the structures of the life-world of the crisis, with the perspective of giving meaning, of helping the subject to re-inscribe the crisis within his history, and to overcome it. Only if the psychiatrist connects with the supporting structures of his own life-world to the life-world of the acute crisis does he have any chance of genuine understanding. This means restoring historical continuity to the crisis, despite its critical discontinuity, and suddenly taking on, during the crisis itself, a perspective that opens to the aftermath of the crisis. Which is ultimately the best way to prevent the crisis itself. The *a priori* aspects of experience, i.e., its conditions of possibility, which precede and structure it, represent the implicit determinants of the crisis, and it is essential for the clinician to make them explicit, to take them into consideration.

### The atmosphere of emergency

The concept of atmosphere, inaugurated by Tellenbach and taken up in recent years by several authors, appears fundamental in understanding the amalgam of emotional tension, haste and immersiveness that characterizes the emergency room environment (Tellenbach, 1968; Griffero, 2016). When we talk about atmospheres we are referring to nuanced situations and almost devoid of objectifiable elements; and we always refer to sensations that characterize a relationship with a specific person and his world (Bohme, 2017; Griffero and Moretti, 2019). It is always found “between,” in the relationship, in the meeting place, and expresses the emotional tones of the situation. In fact, it could be said that the psychiatrist, before empathetically tuning into the patient’s experiences, pathetically tunes into the atmosphere in which he is immersed, in which both are therefore immersed (Bin, 1992).

Erwin Straus distinguished between sympathetic perception of the world and the topographical representation of a given place. On the one side, the sympathetic perception of the world is immediate, made up of profiles, contours, blurred images in which the distinction between Self and world is lost. On the other side, the topographical (geographical) representation of a given place is “gnostic,” which means that the self is constituted as a pole distant from the world (Straus, 1935). We speak of a “situation” in the emergency because it is precisely a question of being-situated, being-thrown into a context (Heidegger, 2008). The real-world experience of emergency is an immersive experience, whose coordinates are often lacking (Barra et al., 2007). The clinician generally intervenes in a drama whose origin he does not know, but he is involved in the outcome. And often the patient is not in such a condition as to be able to provide adequate information (Meyers and Stein, 2000).

The encounter in the acute crisis takes place like an immersion in a “landscape” of which it is difficult to decode everything. But in that chaos we grasp some things, or *almost-things*, in an immediate and sympathetic relationship (Schmitz, 2019). The experience of emergency is in fact detached from daily life of our being-in-the-world, both from the clinician’s side and from that of the patient. From the patient’s perspective, the risk is to lose himself in this pathetically charged and tense atmosphere: that’s why he needs someone to walk alongside him to find the reins of his world. The clinician who intervenes is himself “in” the crisis, therefore immersed in the landscape of the crisis, yet at the same time he is “faced with” the crisis and maintains the ability to abstract coordinates such as to trace his position and that of the patient with respect to a context. The context of the emergency room puts the clinician in the position of applying Strauss’s sympathetic perception of the world, made up of atmospheres, sensations, profiles, and not only of eidetic knowledge (Straus, 1935).

The first knowledge that the clinician has of the psychiatric emergency is therefore pre-conceptual, lightning fast. The situation is perceived “as a whole,” going beyond the details and particularities as a function of a glance at the whole. And it is not just a matter of looking at the patient, but at the clinician himself, who through his emotions can be directed to understand the patient’s life-world (Ballerini, 2003). For example, we speak of “praecox feeling” to indicate that difficult to circumscribe sensation that arises in front of a psychotic person, the sensation of not being able to communicate, of speaking two different languages (Ruemke, 1948). They are not only counter-transferential elements, but resonances of the environment that are captured and experienced by the clinician who is immersed in them.

The psychiatrist’s main anchor is his own feeling. The acute modification of the psychotic presence is articulated and given as a modification of feeling or as a complete involvement of the patient’s state of consciousness (Conrad, 1958; Baeyer Von, 1966). This involves a radical catastrophe of the patient’s situation of existence and of the relationship with the Other, which shipwrecks simultaneously with the relationship with himself (De Martis et al., 1989). The distress experienced by the patient in the acute state, for example, may be proportional to the degree of stabilization or crystallization of the experience. If the patient experiences a magmatic phase, such as that of the *Wahnstimmung*, his anguish is maximum. If, on the other hand, the patient enters the acute situation already with a productive superstructure (consolidation phase), then his anguish results to be channeled. The very acute phase (*Trema*) is characterized by exponential anguish: the patient experiences a universe that loses ordinary meanings without taking on new ones, and the atmosphere is sinister and full of negative omens (Conrad, 1958). The patient in this phase has a perplexed, hypervigilant or detached attitude, pervaded by restlessness. Growing tension (*Spannungstimmung*), indeterminacy, inexplicability, incomprehensibility are constants, together with the premonition of catastrophe (Callieri, 1955). In this phase the psychiatrist’s attitude must be based on reassurance, at a distance, avoiding starting a dialogue without gravity.

The atmosphere of the emergency room, very emotionally charged and characterized by tension, pressure, urgency, is one of the most difficult to bear. For this reason, it becomes fundamental to describe its coordinates, to make explicit its conditions of possibility. Only in this way will the clinician not be dominated by it, but will be able to maintain a prospective position that allows him not only to stem the crisis within himself, but also to help the patient find his personal way out. An atmosphere that can become oppressive, if not thematized, and that can lead the clinician to defend himself in the haste and superficiality of the intervention.

### The phenomenological method

The phenomenology of the acute crisis therefore unfolds in a limited space–time environment (the emergency room), generally more suitable for organic emergencies, with a trend that presses, like a cone or a funnel, sometimes violently towards the resolution. By definition, it belongs to the temporal structure of what is acute to undergo a rapid evolution. The psychiatrist’s task is therefore threefold: (1) to support the outflow of the crisis; (2) avoid its degeneration by making the patient and bystanders safe; (3) to throw an anchor in the whirlpool to then be able to hook the patient to the treatment. The best strategy should be to keep the patient in treatment even after the acute episode, in order to prevent the onset of another episode, or to attenuate the violence of its manifestation. For the patient, it is important that a maintenance treatment project is already in place, at least within the psychiatrist and the treating team, during the acute phase. At the same time, the clinician must evaluate the space for dialogue with the patient, the context and explosive modalities of the crisis, the pharmacological treatment he has previously undergone. The goal is to understand if and when it is appropriate to practice pharmacological therapy, and finally the opportunity for hospitalization, voluntary or forced.

The quality of the contact that the clinician manages to structure with the patient’s “critical” conscience and with the surrounding alarm is the crux of therapeutic success (Ballerini, 2003). The clinician must never stop thinking, with responsibility, that there is a moment in the negotiation in which the patient gives in, delegates, in some way, beyond the symptoms and beyond appearances. He is asked to trust a stranger, who at that moment perhaps knows more than he does what is happening. If this succeeds, the clinician sees the light at the end of the tunnel. Life can begin again, for him and for the patient, even a minute later, beyond the end of the world.

Psychiatric crisis is always a situation in which we are thrown, perhaps to the highest degree, and the unfolding of references between the Self and the world and between the Self and the Other becomes an essential skill. Even in the absence of an adequate setting, in the intersection between several pressures, the phenomenological method retains its panoramic gaze intact (Stanghellini and Broome, 2014; Stanghellini, 2019). We define it panoramic because it does not aim only at the observation and description of the present phenomena, which are generally characterized by violence, anguish, chaos. It is through the suspension of the *epochè* that the clinician can distance himself from the oppressive atmosphere of the crisis and grasp the coordinates of the patient’s life-world (Husserl, 1962). Only with this attitude does an authentic encounter become possible even in the difficult situation of emergency, paving the way for the challenge of care (Stanghellini, 2016).

## Data availability statement

The original contributions presented in the study are included in the article/supplementary material, further inquiries can be directed to the corresponding author.

## Author contributions

SG and CE: writing and editing. GP: supervision. All authors contributed to the article and approved the submitted version.

## Conflict of interest

The authors declare that the research was conducted in the absence of any commercial or financial relationships that could be construed as a potential conflict of interest.

## Publisher’s note

All claims expressed in this article are solely those of the authors and do not necessarily represent those of their affiliated organizations, or those of the publisher, the editors and the reviewers. Any product that may be evaluated in this article, or claim that may be made by its manufacturer, is not guaranteed or endorsed by the publisher.
